# Integrated microRNA and whole-transcriptome sequencing reveals the involvement of small and long non-coding RNAs in the fiber growth of ramie plant

**DOI:** 10.1186/s12864-023-09711-9

**Published:** 2023-10-09

**Authors:** Yafen Fu, Langbo Yi, Fu Li, Jing Rao, Xiai Yang, Yanzhou Wang, Chan Liu, Touming Liu, Siyuan Zhu

**Affiliations:** 1 Institute of Bast Fiber Crops, Chinese Academy of Agricultural Sciences, Changsha, China; 2 College of Biology and Environmental Sciences, Jishou University, Jishou, China; 3 Yangzhou University, Yangzhou, China

**Keywords:** Ramie, Fiber growth, Small RNA, Long non-coding RNA, MYB transcriptional factor

## Abstract

**Background:**

MicroRNAs (miRNAs) and long non-coding RNAs (lncRNAs) are the two main types of non-coding RNAs that play crucial roles in plant growth and development. However, their specific roles in the fiber growth of ramie plant (*Boehmeria nivea* L. Gaud) remain largely unknown.

**Methods:**

In this study, we performed miRNA and whole-transcriptome sequencing of two stem bark sections exhibiting different fiber growth stages to determine the expression profiles of miRNAs, lncRNAs, and protein-encoding genes.

**Results:**

Among the identified 378 miRNAs and 6,839 lncRNAs, 88 miRNAs and 1,288 lncRNAs exhibited differential expression. Bioinformatics analysis revealed that 29 and 228 differentially expressed protein-encoding genes were targeted by differentially expressed miRNAs and lncRNAs, respectively, constituting eight putative competing endogenous RNA networks. lncR00022274 exhibited downregulated expression in barks with growing fibers. It also had an antisense overlap with the MYB gene, *BntWG10016451*, whose overexpression drastically increased the xylem fiber number and secondary wall thickness of fibers in the stems of transgenic *Arabidopsis*, suggesting the potential association of lncR00022274-*BntWG10016451* expression with fiber growth.

**Conclusions:**

These findings provide insights into the roles of ncRNAs in the regulation of fiber growth in ramie, which can be used for the biotechnological improvement of its fiber yield and quality in the future.

**Supplementary Information:**

The online version contains supplementary material available at 10.1186/s12864-023-09711-9.

## Background

Plant fibers are essential for humans as they are a major source of raw materials for the production of paper, textiles, and composites [1]. In addition, fibers are important for plant growth and development because they provide mechanical support to different organs as well as the entire plant body [2]. Fiber formation has gained attention in studies of model species because of the remarkable roles of fibers within plants and their commercial applications. Plant fibers are a type of sclerenchymatous cells with thick secondary cell walls that are mainly composed of cellulose, hemicelluloses (xylan and glucomannan), and lignin [3]. Therefore, fiber growth is mainly involved in the biosynthesis of secondary cell walls [4]. Numerous enzyme-encoding genes are involved in the biosynthesis of secondary cell walls, participating mainly in cellulose biosynthesis and assembly, glucomannan biosynthesis, lignin biosynthesis and polymerization, and the patterned deposition of secondary cell walls [3]. Expression of these enzyme-encoding genes is coordinated at an appropriate time by an NAC-MYB-based transcriptional network [3, 5]. In *Arabidopsis*, at least 10 NAC genes have been identified as the top-level master switches of this regulatory network who regulate the expression levels of the second-level master switches, MYB46 and MYB83; MYB46/MYB83 subsequently modulates the expression levels of downstream transcription factors, precisely controlling the entire secondary wall biosynthesis process [3]. MYB transcription factors play an important role in the regulation of plant cell wall biosynthesis and can be classified into the following four types based on the number of MYB structural domains: 1R-MYB, R2R3-MYB, R1R2R3-MYB, and 4R-MYB [6–10]. R2R3-MYB is the largest subfamily of MYB transcription factors in plants [10, 11]. Hence, genomic identification of R2R3-MYB transcription factors has been performed in several sequenced plants. To date, 126 R2R3-MYB transcription factors have been identified in *Arabidopsis* [12], 108 in grape [13], 100 in sweet orange [14], and 222 in apple [15] plants.

In addition to genes regulating the growth of plant fibers, non-coding RNAs (ncRNAs) also affect the plant fibers. NcRNAs differ from their corresponding mRNAs because their base sequences do not encode proteins [16]. NcRNAs are generally divided into two classes based on the length cutoff: those with less than 200 nucleotides are usually referred to as short or small ncRNAs, including microRNAs (miRNAs), and those with more than 200 bases are known as long ncRNAs (lncRNAs) [17]. Although ncRNAs do not encode proteins, they play crucial roles in plant growth and development, including fiber growth [18]. Genome-wide expression profiling has identified many differentially expressed non-coding miRNAs and lncRNAs that are potentially involved in the regulatory network of secondary wall biosynthesis in various species [19–22]. Functional characterization of *miR397a* revealed that this ncRNA modulates the secondary wall biosynthesis by targeting two laccase genes, *LAC17* and *LAC4*, which are involved in secondary wall biosynthesis in *Citrus* [23]. In *Arabidopsis*, *TCP4* promotes, whereas non-coding *miR319* inhibits the biosynthesis of secondary cell wall by targeting *TCP4* [24].

Ramie (*Boehmeria nivea* L. Gaud) is one of the oldest fiber crops in China, with a cultivation history of 4,700 years in the country [25]. Ramie fibers extracted from the stem bark possess many excellent characteristics [26]. Although significant efforts have been made to understand the mechanisms underlying fiber growth [27–31], the basic mechanisms of miRNAs and lncRNAs involved in secondary cell wall biosynthesis in the fibers of many species, especially those of ramie plant, remain unclear. Therefore, in this study, we characterized and compared the expression profiles of miRNAs and lncRNAs between the bast barks of the top stem (TPS) and middle stem (MPS) sections, where the secondary cell walls of fibers are thick and do not initiate plant growth [21], to identify the ncRNAs associated with fiber growth. Our study provides an important basis for understanding the involvement of ncRNAs in the regulation of ramie fiber growth.

## Results

### Characterization of miRNA expression profiles in the bast barks

**Fig. 1:**
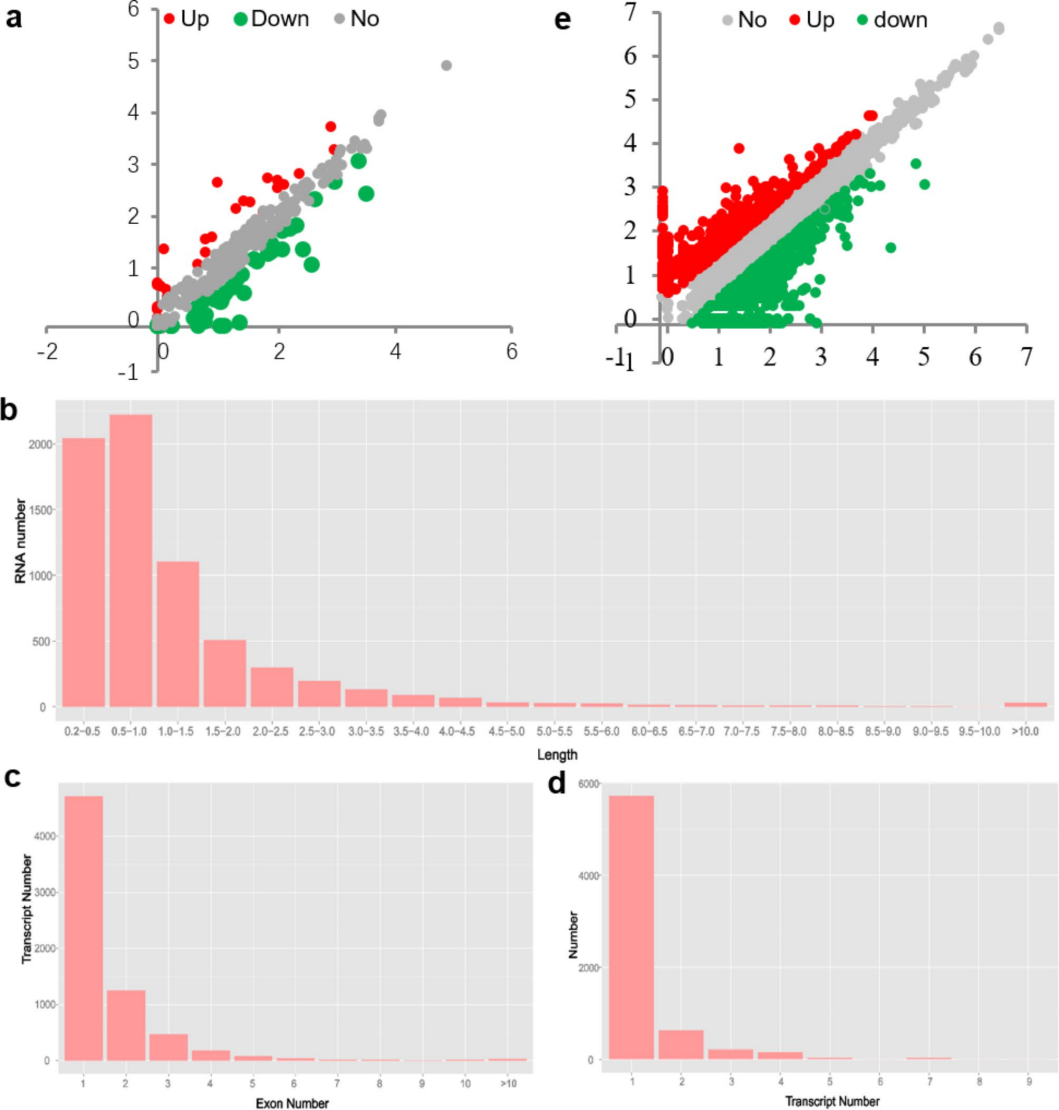
Characterizing the miRNA and lncRNA in the stem barks of ramie. Comparison of the expression level of miRNAs (**a**) and lncRNAs (**e**) between TPS and MPS libraries. Red dots represent transcripts more prevalent in the MPS library, green dots show those present at a lower frequency in the MPS library, and grey dots indicate transcripts that did not change significantly. **b**-**d**, Distribution of lncRNAs length (**b**), exon number (**c**), and transcript number (**d**)

Six bark tissues (three from TPS and three from MPS) were used for small RNA sequencing and yielded a total of 189.0 million clean reads (Table 1). Of these reads, 170.3 million were aligned with known miRNAs by searching a small RNA database, leading to the annotation of 176 ramie miRNAs, and the remaining reads were used for predicting miRNAs, leading to the identification of 202 novel miRNAs (Table S1). Correlation analysis of miRNA expression levels among the six samples revealed a distinct difference between TPS and MPS sample (Fig. S1). Therefore, we compared the expression levels of these 378 miRNAs between the TPS and MPS tissues. The results revealed that 51 and 37 miRNAs exhibited downregulated and upregulated expression, respectively, in fiber-growing MPS tissues (Fig. 1a; Table S2). Five differentially expressed miRNAs were randomly selected for real-time quantitative polymerase chain reaction (qPCR) analysis and their expression differences were further verified (Fig. S2). The results indicated that the differential expression detected using small RNA sequencing was reliable. These differentially expressed miRNAs may play important roles in controlling ramie fiber growth.

**Table 1:** Statistics of reads from small RNA and mRNA sequencing

Sample	Small RNA sequencing		Whole-transcriptome sequencing	
	Clean reads	mapped	Clean reads	mapped
MPS1	31,414,380	29,391,618	152,009,656	119,859,614
MPS2	31,893,369	28,414,895	152,510,394	115,785,891
MPS3	32,372,805	29,007,238	151,294,888	112,260,807
TPS1	30,979,716	28,579,251	151,382,004	122,664,838
TPS2	32,788,217	29,011,030	151,229,676	112,288,034
TPS3	30,419,934	25,898,874	151,692,564	112,540,713
Total	189,868,421	170,302,906	910,119,182	695,399,897

### Identification of differentially expressed lncRNAs and protein-encoding genes between TPS and MPS via whole-transcriptome sequencing

Whole-transcriptome sequencing is a high-efficient tool for the identification of both protein-encoding genes and ncRNAs [32]. In this study, we performed whole-transcriptome sequencing of barks from TPS and MPS, yielding 910 million sequence reads from libraries of six samples (Table 1). These reads were assembled by aligning them with the ramie genome. After predicting the protein-coding potential of the assembled transcripts, 6,839 lncRNAs were identified in ramie (Fig. S3). The length of the lncRNAs ranged from 200 to 76,889 bp, with an average length of 1217.2 bp, which is far smaller than the length of the protein-encoding transcripts (Fig. 1b) [31]. Only a few exons were observed in the identified lncRNA-transcribing genes (average 1.6 exons), and 5,968 of the 6,839 lncRNA-transcribing genes (~ 87.2%) had only one or two exons (Fig. 1c), indicating that fewer exons existed in the lncRNA-transcribing genes than in the protein-encoding genes (~ 6.99 exons) [31]. Of these lncRNA-transcribing genes, 1,107 could yield more than one transcript because of alternative splicing (Fig. 1d). Cluster analysis indicated a distinct difference in the expression levels of these lncRNAs between TPS and MPS samples (Fig. S4). Of these, 1,288 showed differential abundance in the barks of TPS and MPS tissues (Fig. 1e; Table S3). We also randomly selected eight lncRNA-transcribing genes for real-time qPCR analysis and further validated their differences in expression (Fig. S5).

Sequence reads from whole-transcriptome sequencing were used to quantify the expression levels of ramie genes. A distinct difference in gene expression levels between TPS and MPS was observed (Fig. S6). After comparing the transcriptional abundance of ramie genes between the two investigated tissues, 3,308 genes were identified with differential expression (Fig. S7; Table S4). Cellulose is one of the most important components of secondary cell walls, and its biosynthesis is catalyzed by cellulose synthase. There were ten cellulose synthase genes with expression changes in fiber-growing MPS tissues (Table S5), including *BntWG10024039*, an orthologous gene of *Arabidopsis* IRX1 [33]. Notably, these differentially were significantly enriched in Gene Ontology (GO) terms related to secondary cell wall biosynthesis (*P* < 0.01; Fig. S8), including cell wall (GO:0005618), cellulose synthase activity (GO:0016759), and cell wall organization or biogenesis (GO:0071554).

### Potential roles of miRNAs in fiber growth

**Fig. 2:**
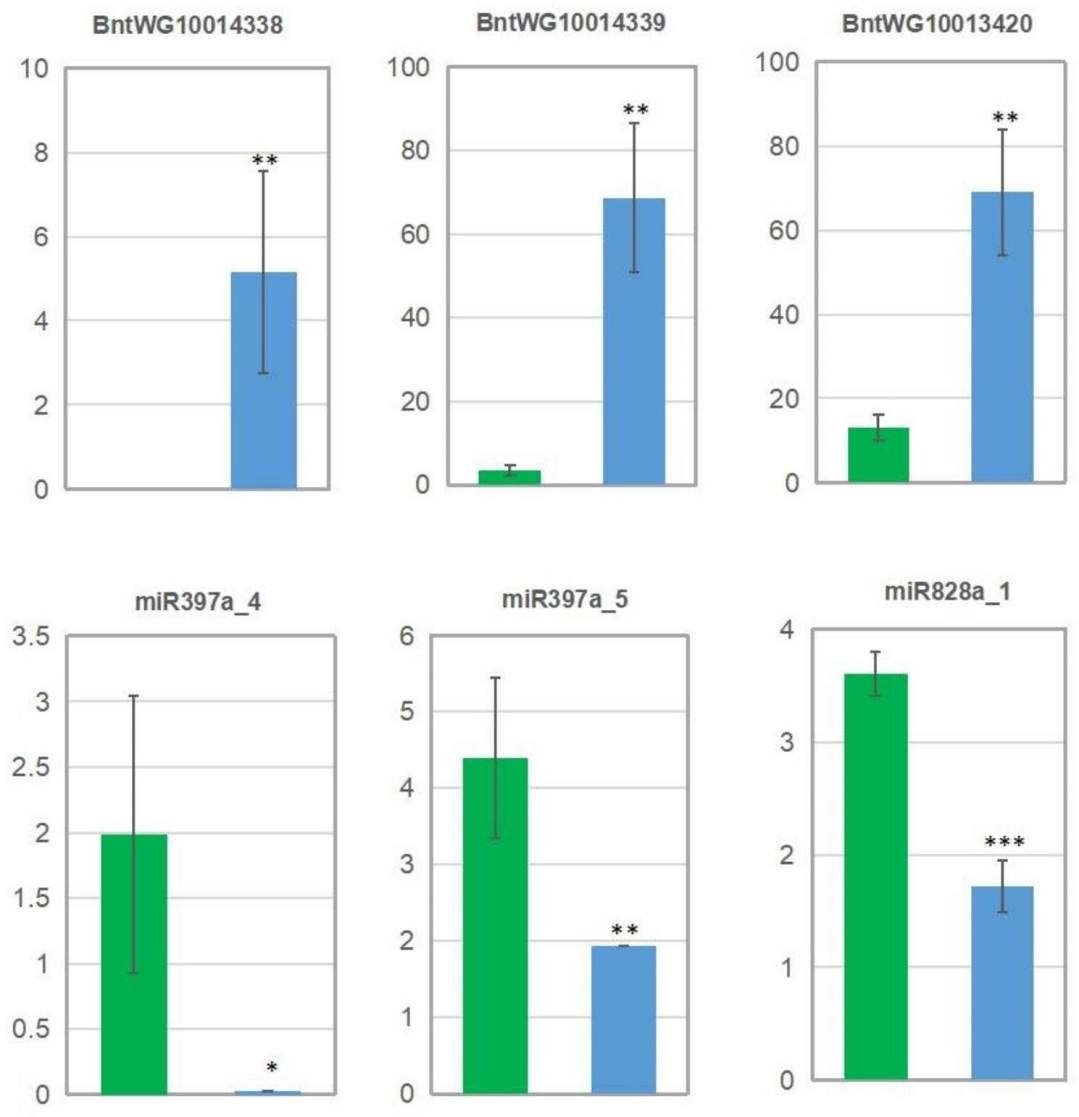
Comparison of expression level of three miRNAs and their targets in TPS and MPS. The y axis of above three figures indicates the FPKM values, while that of above three figures represents TPM value. The green and blue column represents the value of TPS and MPS, respectively. *, **, and *** indicates that the value of MPS has significant difference comparing with that of TPS at the level of 0.05, 0.01, 0.001, respectively

**Fig. 3:**
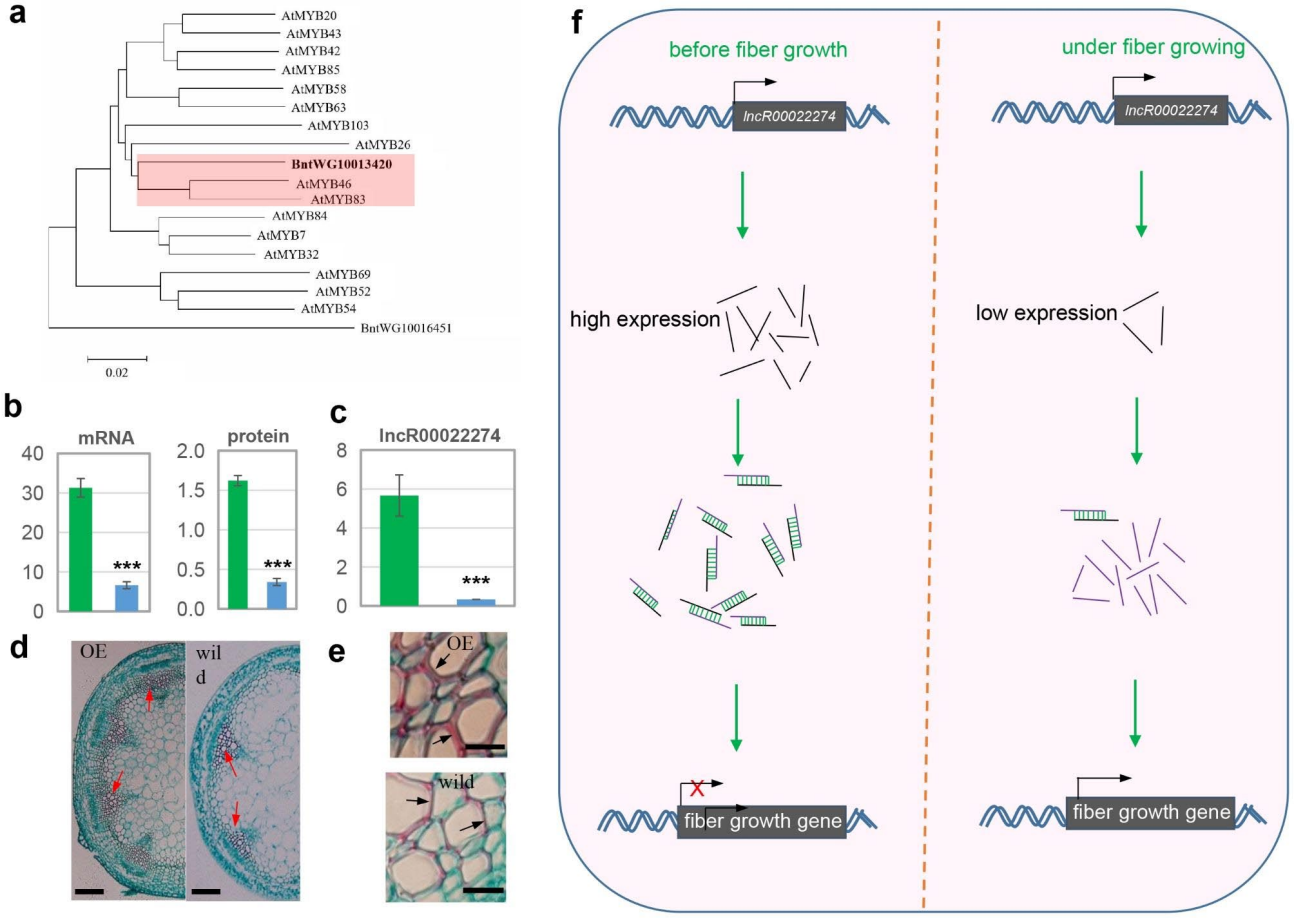
Evidence for the involvement of lncRNA in fiber growth. **a**, Phylogenetic tree of two ramie MYB proteins and Arabidopsis secondary wall-biosynthetic MYB proteins. **b**, Amount of mRNA and protein abundance of *BntWG10008444* between bark from top (TPS; green) and middle (MPS; blue) portions of stems significantly differed between the two tissues (*P* < 0.001). **c**, Significant difference in the FPKM value of lncR00022274 was observed between the TPS (green) and MPS (blue) (*P* < 0.001). **d**, **e**, Light microscopy findings of transected stems of wild and *BntWG10016451-*overexpressing *Arabidopsis*. Arrows in **d** and **e** indicates the cell wall of fiber cells and xylem regions, respectively. Scale bar = 20 μm (d) and 200 μm (e). **f**, A possible model for explaining the function oflncR00022274. The black and purple short lines represent the transcript oflncR00022274 and its target *BntWG10016451*. Before fiber growth, numerous transcripts of lncR00022274 anti-overlapped with the MYB gene *BntWG10016451*, thereby to inhibit the translation activity of *BntWG10016451*

Bioinformatic prediction of the targets of miRNAs identified 683 interacting pairs, consisting of 266 miRNAs and 101 protein-encoding genes (Table S6). Of the 101 protein-encoding targets, 89 were targeted by differentially expressed miRNAs. To explore the role of differentially expressed miRNAs in fiber growth, we investigated the expression profiles of their protein-encoding targets in the stem bark and identified 29 protein-encoding targets with altered expression in the fiber-growing bark (Table S7), including two laccase-encoding genes, *BntWG10014338* and *BntWG10014339*. Laccase is a pivotal enzyme that catalyzes the polymerization of monolignols and thus plays an important role in secondary wall biosynthesis during fiber growth [34, 35]. There was significant upregulation in the expression of *BntWG10014338, BntWG10014339* and *BntWG10013420* in the bark of fiber-growing MPS (Fig. 2), indicating their potential role in fiber growth. Notably, both were targeted by miR397a_4, miR397a_5, and miR828a_1, and three of these miRNAs were downregulated in the fiber-growing MPS (Fig. 2). It is likely that the downregulation of miR397a_4 and miR397a_5 caused the accumulation of transcripts of *BntWG10014338* and *BntWG10014339*, thereby promoting fiber growth. In addition, *BntWG10013420* is an ortholog of *AtMYB46* and *AtMYB83*, which are the two master switches for *Arabidopsis* secondary wall biosynthesis (Fig. 3a). We observed that the transcript abundance of *BntWG10013420* was higher in the libraries of MPS than in those of TPS (Fig. 2), which was potentially caused by the downregulated expression of miR828a_1 (Fig. 2), an miRNA with the putative ability to target the transcripts of *BntWG10013420* and to degrade them.

### Potential roles of lncRNAs in fiber growth

To understand the function of the identified lncRNAs, we performed bioinformatic prediction of their targets. The results revealed that 1,877 lncRNAs were found to target 2,123 ramie genes, leading in 2,630 interacted pairs (Table S8). Of these interactions, 668, 367, and 1,595 were targeted via the trans-, cis-overlap, and cis-non-overlap. To identify the lncRNAs involved in fiber growth, we investigated the targets of differentially expressed lncRNAs and identified 228 targets with altered expression in MPS (Table S9), including IRX15L-homologous *BntWG10008444*. IRX15L encodes a DUF579-containing protein involved in xylan deposition in the secondary wall [36]. A recent proteomic study revealed that the protein abundance of *BntWG10008444* was significantly downregulated in MPS (Fig. 3b; Li et al., 2021). In this study, we observed a distinct decrease in the transcript abundance of *BntWG10008444* in the MPS libraries (Fig. 3b), further validating the finding of expression changes from the previous proteome analysis. Notably, *BntWG10008444* was a target of differentially expressedlncR00050912 via antisense overlapping.

MYB transcription factors play crucial roles in the regulation of fiber growth in *Arabidopsis* [3]. We identified the MYB gene *BntWG10016451* which was targeted bylncR*00022274*, using an antisense overlapping method. Overexpression of this MYB gene caused a significant increase in fibers and drastically thickened the secondary wall of fibers in transgenic *Arabidopsis* (Fig. 3c and d), indicating its potential role in ramie fiber growth. Notably, significant downregulation in the expression oflncR*00022274* was observed in the fiber-growing MPS (Fig. 3e).lncR*00022274* potentially played a role in fiber growth by interacting with *BntWG10016451* (Fig. 3f); this lncRNA predicted fiber growth by recruiting and silencing the transcript of *BntWG10016451*, whereas in MPS, with growing fibers, the expression of this lncRNA was downregulated, thereby activating *BntWG10016451* to promote fiber growth in ramie.

### Identification of the competing endogenous RNA (ceRNA) network associated with fiber growth

**Fig. 4:**
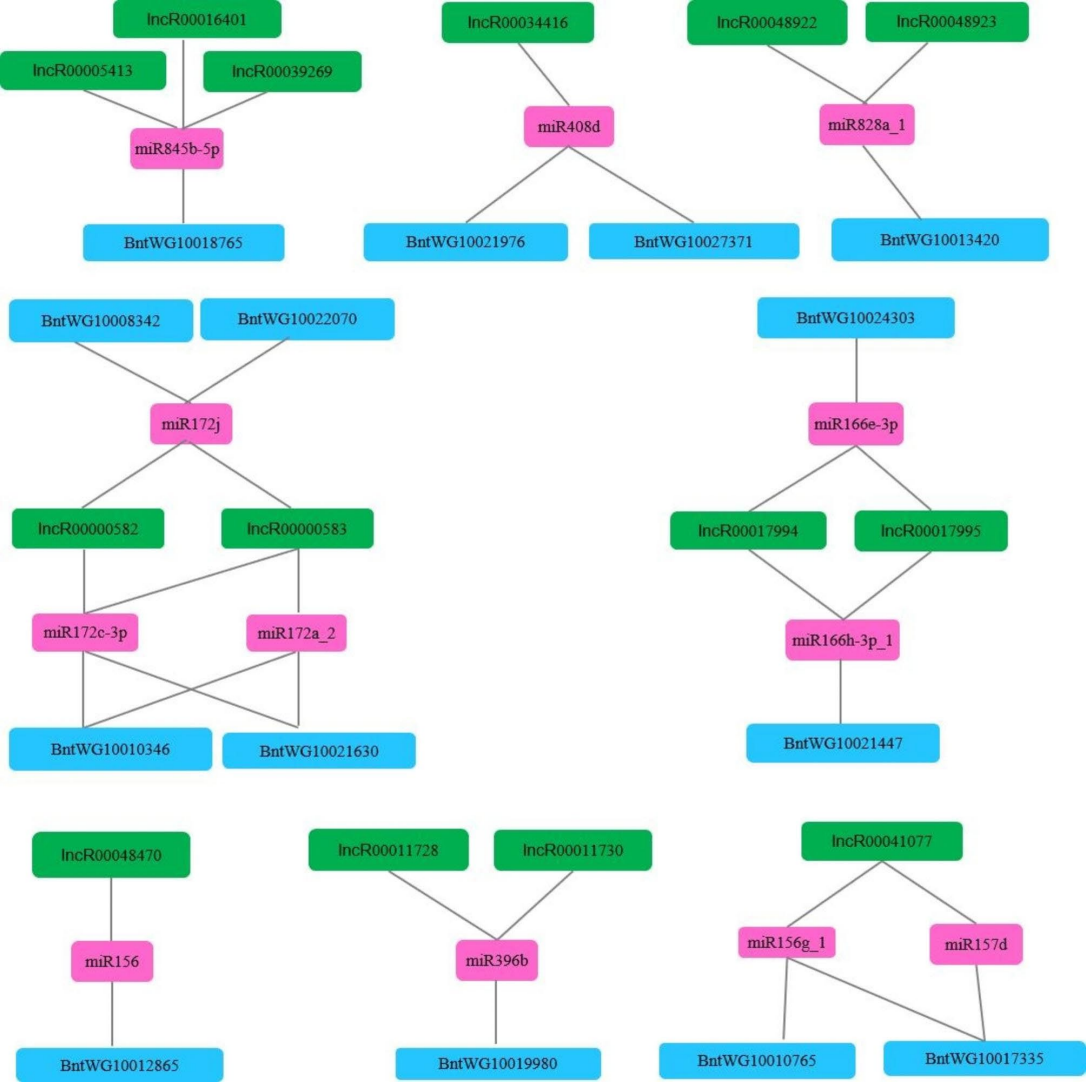
Putative competing endogenous RNA network consisting of 12 differentially expressed miRNAs (pink), 17 differentially expressed lncRNAs (green), and 16 differentially expressed genes (blue)

Bioinformatics analysis of the interactions between miRNAs and lncRNAs revealed that 131 miRNAs targeted 561 lncRNAs, leading to 866 interacting pairs (Table S10). If miRNAs can target both protein-encoding mRNA and lncRNAs, competition for miRNAs between lncRNAs and mRNA will lead to the formation of a ceRNA network. To identify putative ceRNA networks involved in fiber growth, we identified interacting pairs consisting of differentially expressed miRNAs, lncRNAs, and protein-encoding mRNAs. Finally, we identified eight ceRNAs consisting of 12 differentially expressed miRNAs, 14 differentially expressed lncRNAs, and 14 differentially expressed protein-encoding genes (Fig. 4), indicating that these ceRNAs are associated with fiber growth in ramie. Interestingly, *BntWG10013420*, an orthologous gene of *Arabidopsis* secondary wall biosynthetic *AtMYB46* and *AtMYB83* genes, was associated with one of the ceRNAs.

## Discussion

Numerous efforts have been made to determine the fiber growth mechanism [21, 27, 29, 30], which has greatly improved our understanding of its genetic regulation and lead to the establishment of a putative model for fiber formation [28]. Protein post-translational modifications, such as phosphorylation and ubiquitination, are involved in fiber formation [35, 37], indicating a complex mechanism involved in fiber growth. Although differentially expressed miRNAs and circular RNAs have been identified in the stem bark of growing fibers in ramie [20, 37], the specific roles of these ncRNAs and their targets remain unknown.

In this study, we systemically characterized the expression profiles of miRNAs, lncRNAs, and protein-encoding genes in the fiber-growing bark of stems and identified 29 and 228 protein-encoding genes potentially associated with fiber growth that were targeted by differentially expressed miRNAs and lncRNAs, respectively. For example, laccase is one of the most important enzymes for fiber growth owing to its involvement in secondary wall biosynthesis [34, 35]. Two *Arabidopsis* laccase genes, *LAC17* and *LAC4*, are targeted by *miR397a* to mediate their functions. Similarly, this study identified two laccase genes with expression changes in the fiber-growing bark, suggesting their involvement in ramie fiber growth; these genes were targeted by two differentially expressed ramie miRNAs, *miR397a_4* and *miR397a_5*. Additionally, we identified a differentially expressed gene that was an ortholog of secondary wall-biosynthetic *IRX15L* [36], which was targeted by differentially expressedlncR*00050912*. Whole-transcriptome sequencing revealed the *BntWG10013420* gene, which has a similar DNA-binding and repression structural domain as the amino acids of the *Arabidopsis AtMYB4* gene. In contrast, the *Arabidopsis AtMYB4* gene has an inhibitory effect on secondary wall biosynthesis, particularly in the phenylpropane synthesis pathway of *Arabidopsis* thaliana, blocking lignin biosynthesis [38]. The same phenotype was observed with the overexpression of *TaMYB1D* [39] and *TaMYB4* [40] in wheat, *PvMYB4* [41] in willowherb (*Panicum virgatum*), and *PtMYB156* [42] in poplar, all of which negatively affect secondary cell wall biosynthesis. Loss of function of the rice (*Oryza sativa*) MYB4 transcription factor (Os MYB108) [43] favors lignification. Furthermore, we hypothesized that the *BntWG10013420* gene may be a candidate transcription factor that inhibits secondary wall biosynthesis in ramie. Therefore, this study provides distinct evidence supporting the involvement of miRNAs and lncRNAs in ramie fiber growth.

MYB transcription factors play pivotal roles in triggering secondary wall biosynthesis, and at least 16 MYB proteins have been reported to be involved in the regulatory network of secondary wall biosynthesis in *Arabidopsis thaliana* [3]. *Whole _GLEAN_10015497* is a ramie MYB gene associated with fiber growth, and its overexpression causes a significant increase in the numbers of xylem and phloem fibers and thickening of the secondary wall in stem fibers [44]. Interestingly, a previous study revealed that the function of *BntWG10015497* was modulated via ubiquitin modifications; that is, when fibers are growing, a significant reduction in the ubiquitination levels of this MYB protein results in its accumulation [44]. In this study, we identified another MYB gene, *BntWG10016451*, that was associated with the fiber growth of ramie. It was targeted by lncR*00022274* due to an antisense overlap in their sequences, suggesting that the function of *BntWG10016451* is potentially mediated by this lncRNA. These findings indicate that both lncRNA and ubiquitin modifications are involved in the modulation of MYB gene functions, suggesting a complex mechanism for fiber growth.

## Conclusions

In this study, we identified 88 miRNAs and 1,288 lncRNAs exhibiting differential expression between the TPS and MPS tissues. These differentially expressed miRNAs and lncRNAs were predicted to target 29 and 228 differentially expressed protein-encoding genes, respectively, resulting in eight putative ceRNA networks. lncR00022274, an lncRNA with downregulated expression in MPS, exhibited antisense overlap with the MYB gene, *BntWG10016451*. Notably, overexpression of this MYB gene drastically increased the xylem fiber number and thickened the secondary cell wall of fibers in transgenic *Arabidopsis*, suggesting the potential role of thelncR00022274-BntWG10016451 module in ramie fiber growth.

## Methods

### Experimental material and RNA isolation

Zhongzhu 1, an elite cultivar that is widely cultivated in China, was selected as the experimental material for this study. Seedlings were planted in the experimental farm of the Institute of Bast Fiber Crops, Chinese Academy of Agricultural Sciences (Yuanjiang, China) in June, 2016. In 2018, according to the description of Li et al. [37], sections from the top and middle parts of the stems of plant (30-day-old) were separately collected, mixed, and used as TPS and MPS samples, respectively. Three biological replicates of TPS and MPS were collected and immediately frozen in liquid nitrogen. Total RNA was extracted using the E.Z.N.A. Plant RNA kit (OMEGA Bio-Tek, Norcross, GA, USA), according to the manufacturer’s protocol.

### Library construction and sequencing

Total RNAs from six samples were used to construct the small RNA and cDNA libraries. For whole-transcriptome sequencing, cDNA libraries containing both mRNAs and lncRNAs were constructed using RNAs, except ribosomal RNAs. Small RNA sequencing was performed using the BGISEQ-500 platform (BGI, Shenzhen, China), and whole-transcriptome sequencing was performed using the Illumina sequencing platform (HiSeq 2500; Illumina, San Diego, CA, USA), according to the manufacturers’ instructions. The raw reads for each sample were filtered to generate clean reads for further analysis.

### Transcriptome assembly and protein-coding potential analysis

Clean reads sequenced from the cDNA libraries were aligned to the ramie genome (accession ID: PRJNA663427) using the HISAT software [45]. StringTie program [46] was used to assemble the transcripts with default parameters. The protein-coding potential of the transcripts was determined using four approaches: alignment to the PFAM database (default parameters) [47] and prediction using the CPC software [48], txCdsPredict (http://hgdownload.soe.ucsc.edu/admin/jksrc.zip), and CNCI [49]. Score thresholds were set to distinguish lncRNAs from mRNAs for the three programs: 0 for CPC and CNCI and 500 for txCdsPredict. Transcripts reported as lncRNAs using at least three of the four prediction methods were confirmed as lncRNAs.

### Prediction of miRNAs

To identify miRNAs, clean reads from small RNA sequences were aligned to the miRbase [50] and Rfam [51] databases using the AASRA [52] and CMsearch software [53], respectively. Reads unaligned to these two databases were used to predict novel miRNAs using PIPmiR [54] with default parameters. To determine the homologs of our selected candidates from *Arabidopsis* fiber growth-related MYB/NAC proteins, protein alignment was carried out using the Clustal program [55], and an unrooted phylogenetic tree was constructed using the MEGA 5 software with the neighbor-joining method and a bootstrap test with 1000 replicates [56].

### Prediction of miRNA and lncRNA targets

The miRNA targets were predicted using TAPIR [57] and Target Finder [58] with default parameters. Only the targets identified by both tools were considered. LncRNAs execute their functions by targeting mRNAs in cis- or trans-manner [59]. Therefore, to predict the targets of lncRNA, Spearman’s and Pearson’s correlation coefficients were estimated between protein gene and lncRNA, and only the lncRNA–protein gene pair with spearman_cor ≥ 0.6 and pearson_cor ≥ 0.6 was considered for subsequent analyses. If the lncRNA and protein gene were co-located in a 10-kb region, the lncRNA was deemed to target the protein gene via cis-regulation. Otherwise, the RNAplex software was used to analyze the trans-acting targets [60], with parameters ‘−e − 30.’ Functional analyses of the lncRNA target genes were conducted using Blast2GO [61]. GO terms with P-value < 0.05 were considered to be enriched [62].

### Differential expression analysis

To estimate the expression levels of protein-encoding genes and lncRNAs, clean reads from transcriptome sequencing were aligned to the ramie genome (accession ID: PRJNA663427) using the Bowtie2 software [63], and the read number of each transcript was calculated using the RSEM software [64]. Fragments per kilobase of transcript sequence per million base pairs sequenced (FPKM) values were estimated to measure the expression level of each transcript [65]. For miRNAs, the number of clean tags for each miRNA from small RNA sequencing was calculated and normalized to the number of transcripts per million clean tags (TPM) to measure the expression level of each miRNA [66]. Differentially expressed genes, lncRNAs, and miRNAs were detected using the DEGseq program based on the MA-plot algorithm [67]. To identify the expression differences, *P*-values adjusted (Q value) for multiple testing using the Benjamini–Hochberg procedure [68] were used to control for false-positives. Sequences were deemed to be significantly differentially expressed if Q value < 0.05 and at least a two-fold change was obtained by dividing the average FPKM/TPM value of the three MPS libraries by that of the three TPS libraries.

### Overexpression of *BntWG10016451*

To confirm the functions of *BntWG10016451*, its full-length cDNA was amplified using high-fidelity thermostable DNA polymerase, with the following the primer sequences: 5′-ATGACAAGAGACCCAAAGCCAA-3′ (forward primer) and 5′-TCTTCTGAAAGAAAGCATATTTGGC-3′ (reverse primer). Amplified fragments were ligated to the downstream region of the *CaMV 35 S* promoter in PBI121 vector. After confirming proper ligation via Sanger sequencing, 35 S::*BntWG10016451* was introduced into the *Agrobacterium tumefaciens* strain, GV3101, using heat shock, and the resultant *Agrobacterium* was infiltrated into *Arabidopsis* using the floral dip method [69]. Transgenic *Arabidopsis* plants were grown in a growth chamber under the following conditions: temperature, 22 °C; light, 16-h/8-h light/dark cycle. Finally, stem cell tissue sections prepared from 40-days-old transgenic plants were observed under a transmission electron microscope.

### Electronic supplementary material

Below is the link to the electronic supplementary material.

Supplementary Material 1

Supplementary Material 2

## Data Availability

These sequence data have been submitted to the DDBJ/EMBL/GenBank databases under accession number GSE130587. https://www.ncbi.nlm.nih.gov/geo/query/acc.cgi?acc=GSE130587.

## List of abbreviations

ceRNA: competing endogenous RNA
lncRNAs: long non-coding RNAs
miRNAs: microRNAs
