# Supplementary material for: Integrated microRNA and whole-transcriptome sequencing reveals the involvement of small and long non-coding RNAs in the fiber growth of ramie plant
Source: BMC Genomics. 2023 Oct 9;24:599. doi: 10.1186/s12864-023-09711-9 (PMC10563232; doi:10.1186/s12864-023-09711-9)
Supplement: Supplementary file 2 — Supplementary Material 2 [file 12864_2023_9711_MOESM2_ESM.docx]

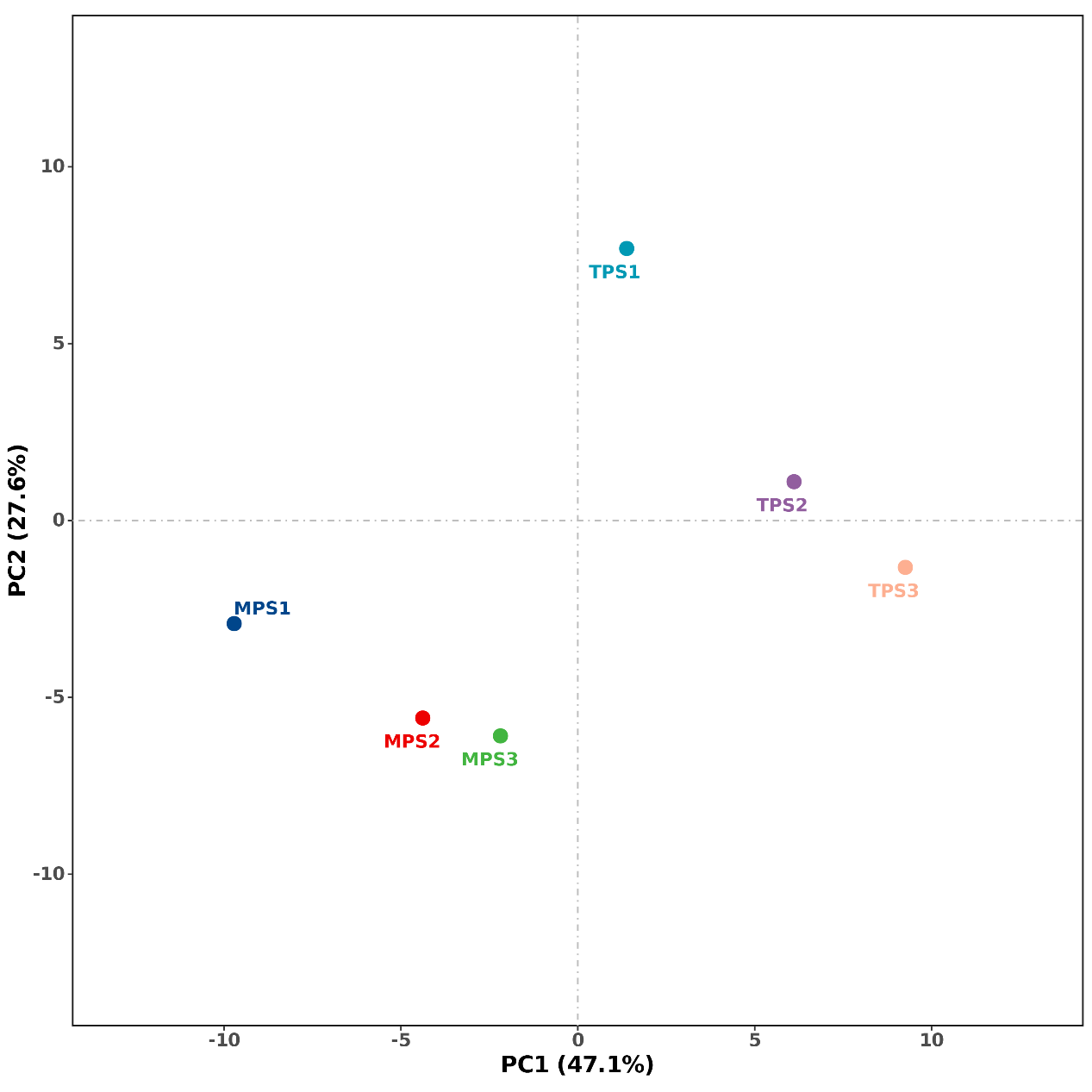


**Fig. S1** Principal commonent analysis for six samples according to the expression level of miRNAs.


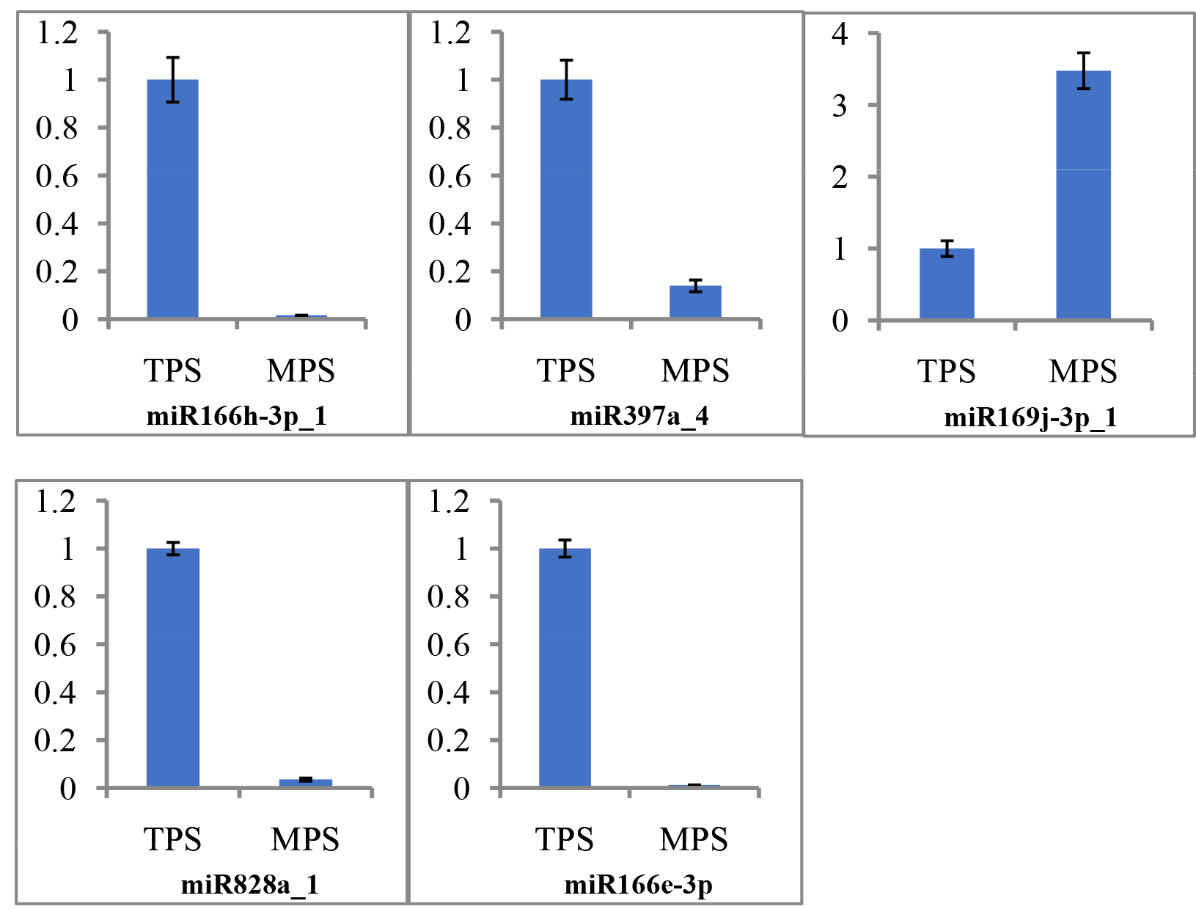


**Fig. S2** qRT-PCR analysis of five differentially expressed miRNAs. Data represent fold change of each gene’s relative quantification in TPS vs.MPS samples; the error bar represented the standard deviation.


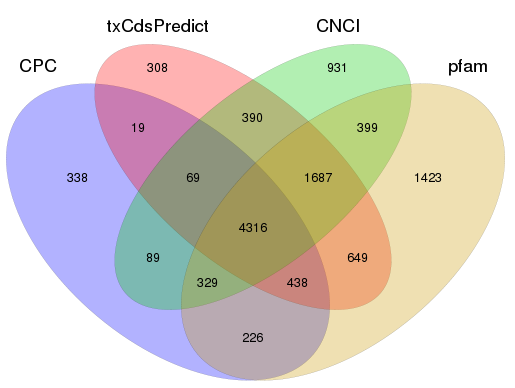


**Fig. S3** Venn diagram showed the transcripts without protein-coding potential based on four bioinformatic methods.


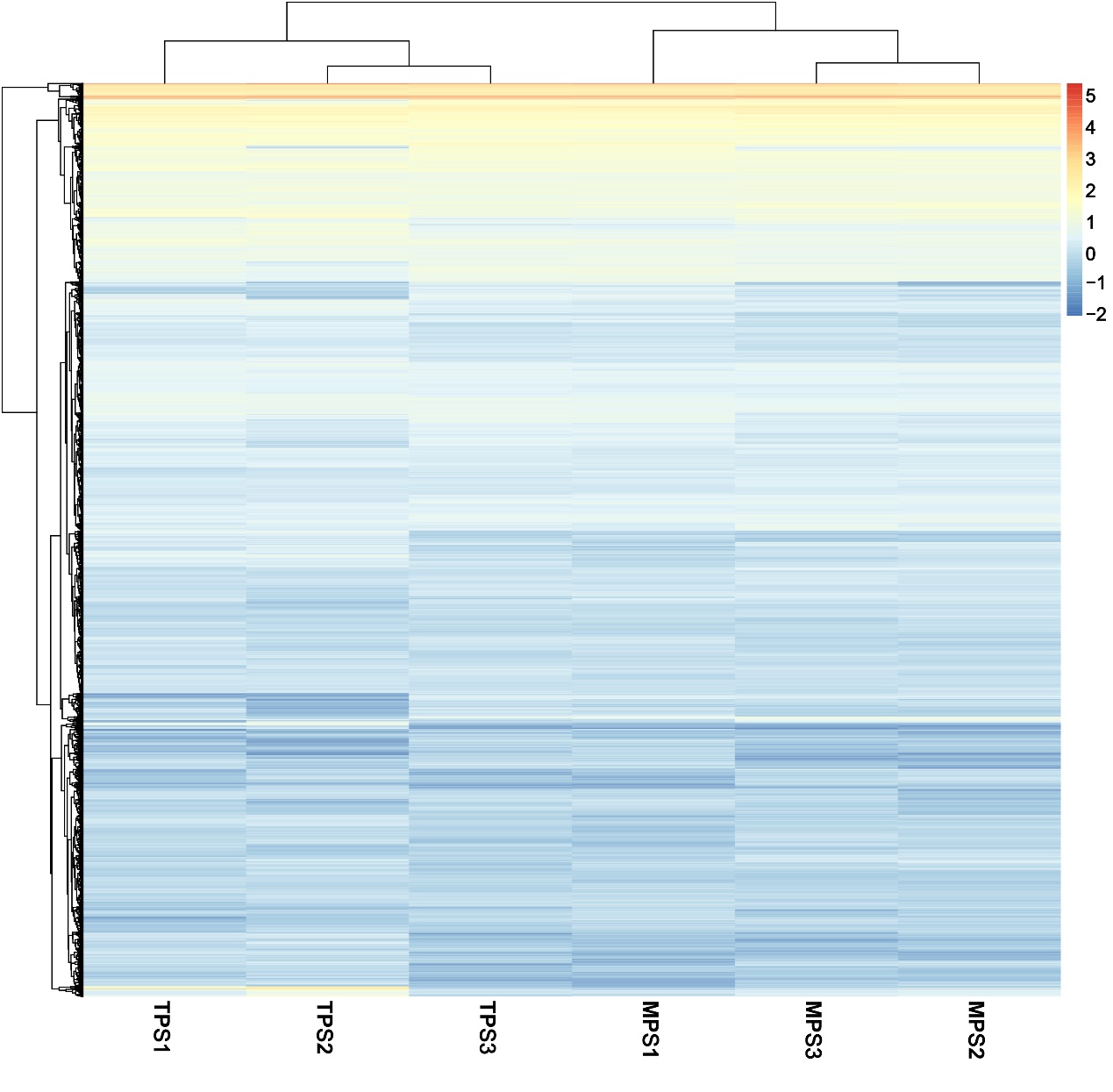


**Fig. S4** The distinct difference in the expression levels of these lncRNAs between TPS and MPS samples


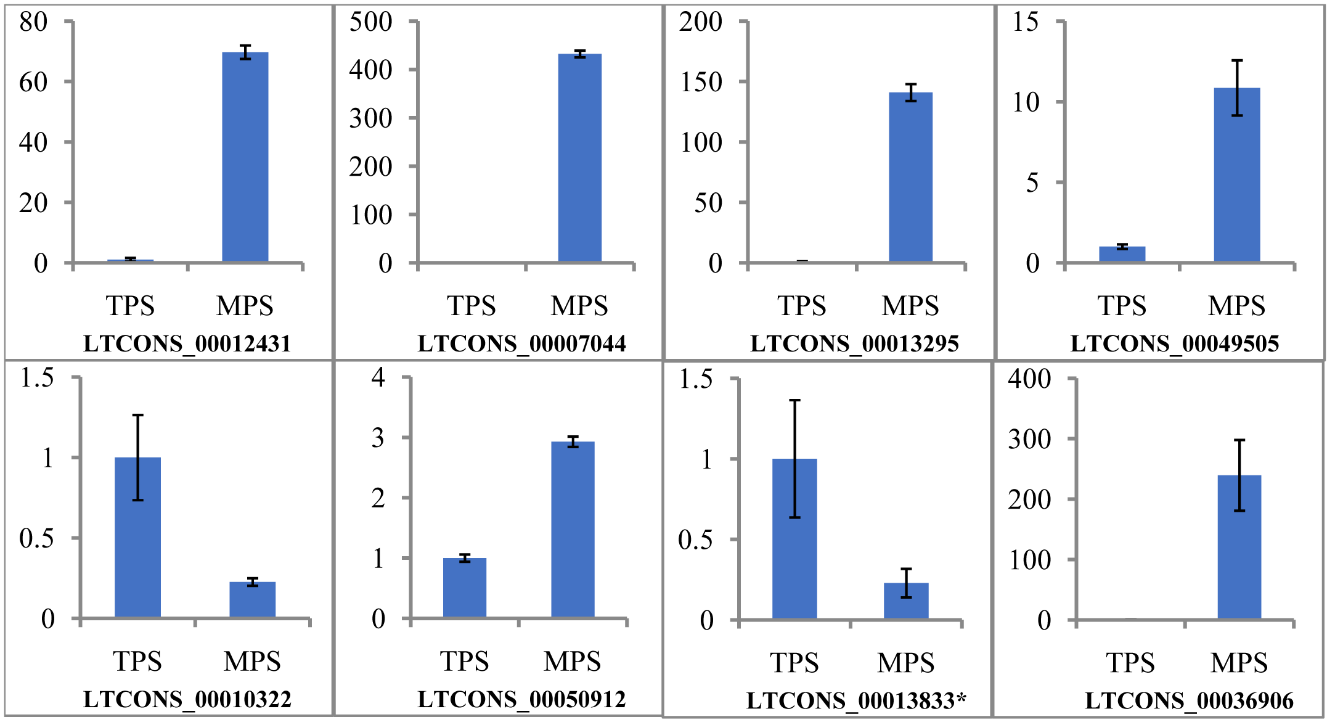


**Fig. S5** qRT-PCR analysis of eight differentially expressed miRNAs. Data represent fold change of each gene’s relative quantification in TPS vs.MPS samples; the error bar represented the standard deviation.


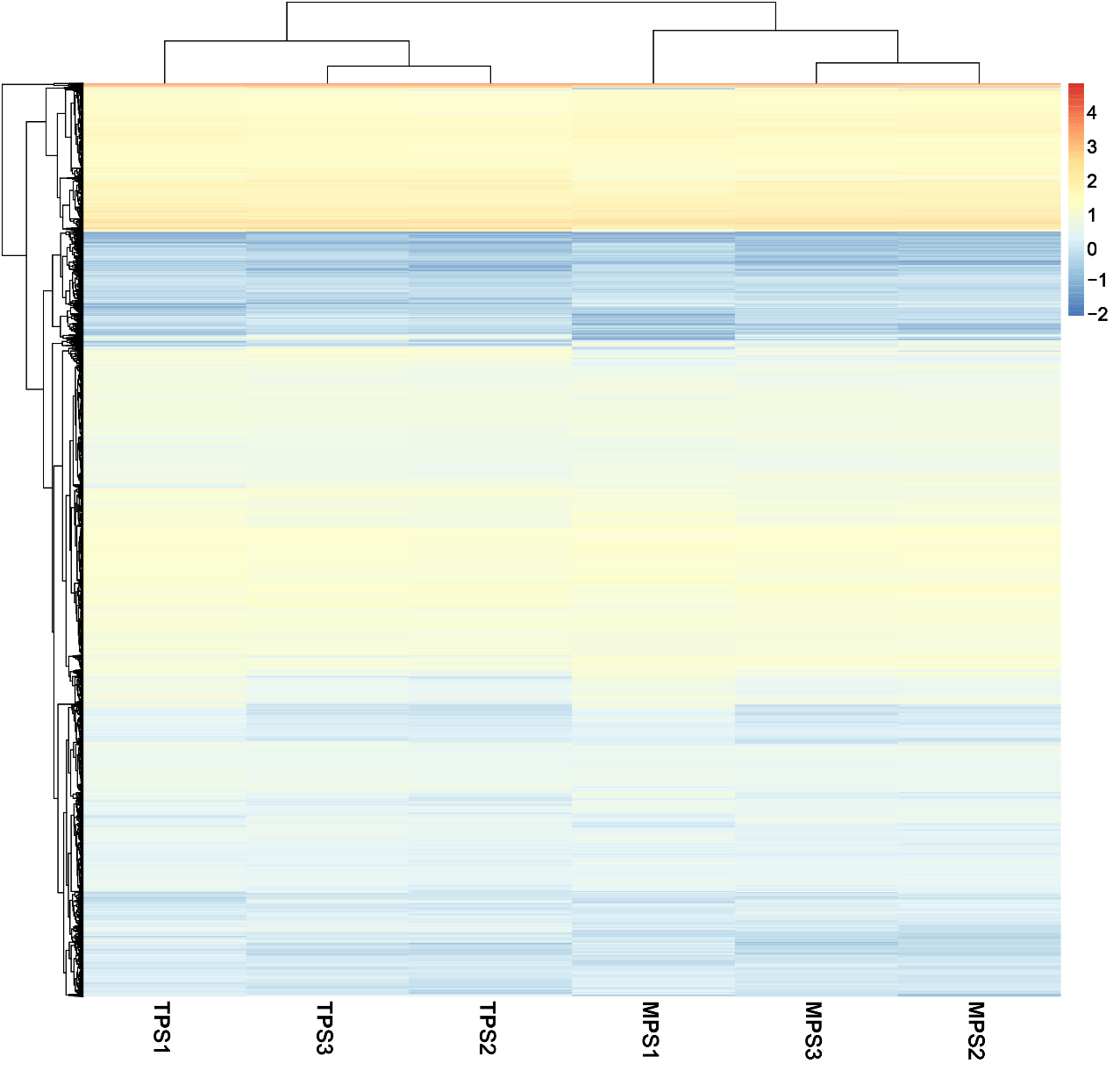


**Fig. S6** The distinct difference in gene expression levels between TPS and MPS.


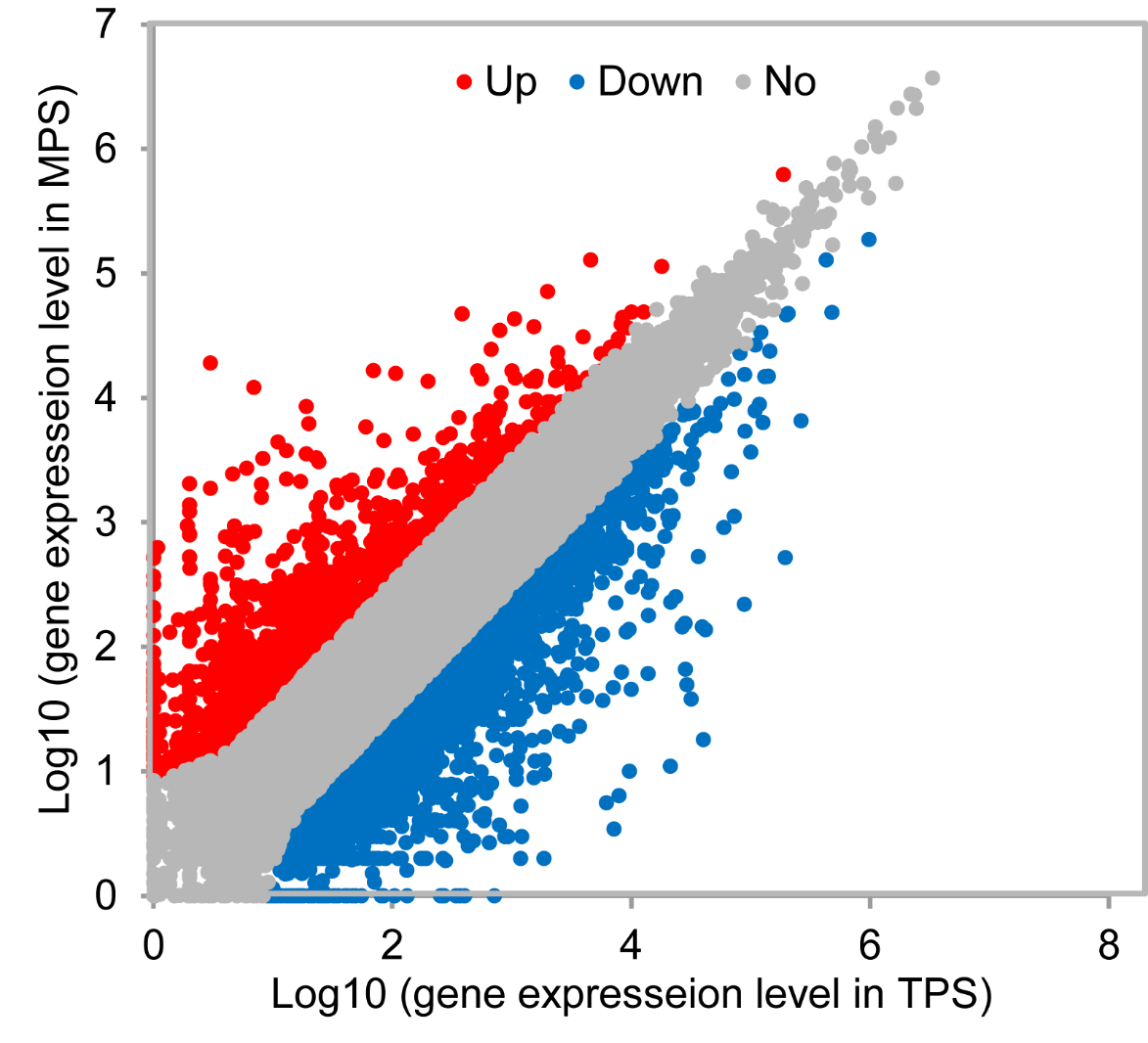


**Fig. S7** Comparison of expression level of protein-encoding genes between TPS and MPS libraries. Red dots represent transcripts more prevalent in the MPS library, green dots show those present at a lower frequency in the MPS library, and blue dots indicate transcripts that did not change significantly.


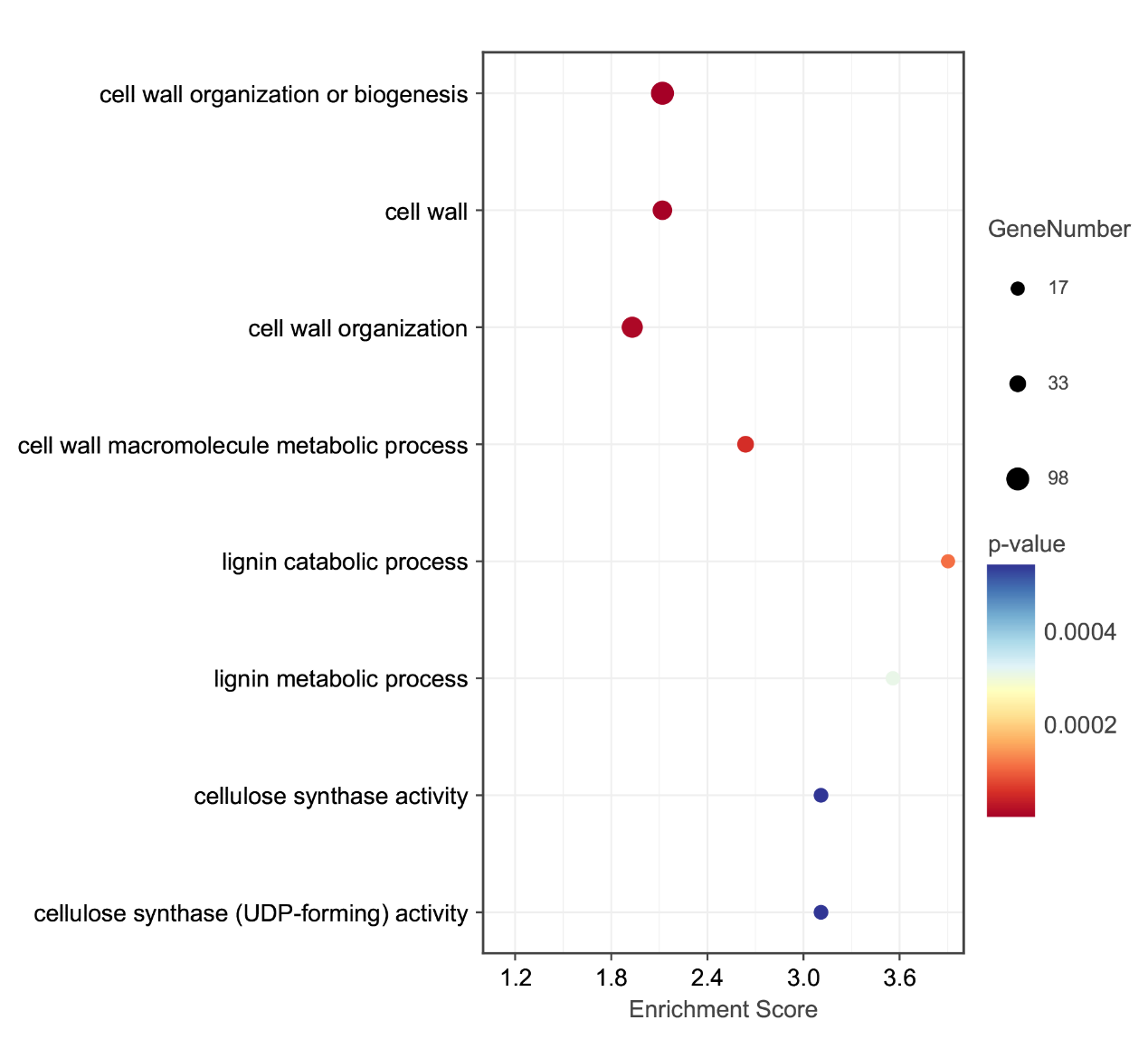


**Fig. S8** Secondary cellular wall biosynthesis-related GO terms with a significant enrichment (*P* < 0.01)
